# Depression, Anxiety and Symptoms of Stress among Baccalaureate Nursing Students in Hong Kong: A Cross-Sectional Study

**DOI:** 10.3390/ijerph13080779

**Published:** 2016-08-03

**Authors:** Teris Cheung, Siu Yi Wong, Kit Yi Wong, Lap Yan Law, Karen Ng, Man Tik Tong, Ka Yu Wong, Man Ying Ng, Paul S.F. Yip

**Affiliations:** 1 School of Nursing, Hong Kong Polytechnic University, Hong Kong, China; 12125573D@connect.polyu.hk (S.Y.W.); 12089424D@connect.polyu.hk (K.Y.W.); 12068084D@connect.polyu.hk (L.Y.L.); 12082685D@connect.polyu.hk (K.N.); 12065489D@connect.polyu.hk (M.T.T.); 12089477D@connect.polyu.hk (K.Y.W.); 12047158D@connect.polyu.hk (M.Y.N.); 2 Centre for Suicide Research and Prevention, University of Hong Kong, Hong Kong, China; sfpyip@hku.hk

**Keywords:** anxiety, DASS 21, depression, epidemiology, stress, mental health education, nursing students

## Abstract

This study examines the prevalence of depression, anxiety and symptoms of stress among baccalaureate nursing students in Hong Kong. Recent epidemiological data suggest that the prevalence of mild to severe depression, anxiety and stress among qualified nurses in Hong Kong stands at 35.8%, 37.3% and 41.1%, respectively. A total of 661 nursing students were recruited to participate in our cross-sectional mental health survey using the Depression, Anxiety and Stress Scale 21. Multiple logistic regression was used to determine significant relationships between variables. Working in general medicine, being in financial difficulty, having sleep problems, not having leisure activity and perceiving oneself in poor mental health were significant correlates of past-week depression, anxiety and stress. Year of study, physical inactivity and family crisis in the past year correlated significantly with depression. Imbalanced diets significantly correlated with anxiety. Stress was significantly associated with a lack of alone time. This is the first study to confirm empirically that clinical specialty, financial difficulties and lifestyle factors can increase nursing students’ levels of depression and anxiety and symptoms of stress. Prevention, including the early detection and treatment of mental disorder, promises to reduce the prevalence of these indicators among this group.

## 1. Introduction

Some students find the transition from adolescence to adulthood stressful. At university, students first start to become responsible for their own life decisions and lifestyle, healthy or otherwise. First-year students need especially to adapt to a new learning environment and cope with academic and social demands of professional training [1]. High academic expectations are stressful and can theoretically in themselves pose risks to students’ physical and mental health [2]. The most common psychiatric problems found among students are depression, anxiety and stress [3,4]. Recent local and international studies reveal a heavy prevalence of depression among freshmen [5,6], besides students in other years of study [7,8]. Ibrahim et al. [8] review 24 studies (*n* = 48,650), including nine from the U.S. and five from East Asia (two from Hong Kong, one from China, and two from South Korea), to reveal a prevalence rate of depression in the samples of between 10% to 85%, with a weighted mean prevalence of 30.6%. Half of these studies explicitly dealt with medical students.

A recent large-scale epidemiological study in China (*n* = 5245) found that 11.7% of university students were depressive. Four percent could be diagnosed as sufferers from Major Depressive Disorder in terms of the Diagnostic Manual of Mental Disorders-Fourth Edition (DSM-IV) [7]. However, despite these alarming findings, there remains a dearth of studies examining the prevalence rates of psychiatric morbidity among ethnically Chinese nursing students in Asia.

Since 2012, the Education Reform was undertaken in Hong Kong and a “334 scheme” was introduced, with three years of junior secondary education and three years of senior secondary education, followed by the Hong Kong Diploma of Secondary Education (HKDSE). Apart from the structural changes in secondary schools, local universities aligned with a curriculum reform, particularly in the bachelor of nursing programs, which has been shifted from a traditional four-year curriculum to five-year curriculum. The newly introduced five-year curriculum has inevitably added extra strain on nursing undergraduates. Unlike other non-nursing undergraduates, nursing students are mandatorily required to have clinical practicum, skills examinations and other course work assignments. The intensive study load alongside with other financial burden, interpersonal relationship problems, adjustment in university life, etc. may place extra strain on baccalaureate nursing students. This psychological burden may proportionally increase the risk of psychiatric morbidity among them. Between October 2015 and March 2016, a surge of 22 student suicides was reported since the start of the academic year. The youth suicide rates double the amount of the average. Student suicides in Hong Kong signaled a significant level of unresolved distress, which should be seriously addressed by mental health experts.

This study is the first ever prevalence study examining levels of depression, anxiety and symptoms of stress among baccalaureate nursing students in a local university in Hong Kong. It is important to examine psychiatric morbidity among university students, since most lifetime mental disorders have their first onset typically when subjects are at college [9]. Understanding university students’ mental health may also have major implications for campus health services and mental health policymaking for this vulnerable group. Furthermore, as nurse are helping professionals we need to understand them better such that they can be better equipped to helping others upon graduation.

## 2. Materials and Methods

### 2.1. Aim

This paper forms part of a large survey-based study of baccalaureate nursing students’ mental health. Specifically, it sets out to examine the weighted prevalence of depression, anxiety and stress among nursing students in the context of a characterization of the socio-demographic characteristics of nursing students in a Hong Kong tertiary institute.

### 2.2. Study Design

This study adopted a cross-sectional design. It took account of existing nursing literature on mental health in drawing up a five-section cross-sectional survey, administered by researchers to nursing students. This paper only reports weighted estimates for depression, anxiety and symptoms of stress as measured by a short version of Depression Anxiety and Stress Scale (DASS 21) [10], and discussed its significant correlates in each dimension.

### 2.3. Participants

The researchers invited the participation of all nursing students registered with the School of Nursing, which offers a five-year curriculum leading to a baccalaureate degree in nursing or mental health nursing. However, the research institution only adopted the new curriculum since 2012 and thus, there were no Year 5 students throughout the data collection period in the academic year 2015–2016. A mass invitation email was delivered to nursing students of Year 1 to Year 4, provided subjects met selection criteria of being aged between 18 and 30, male or female in any clinical specialty and currently registered as full-time students. We further excluded those unable to read Chinese as the Chinese version of the Mental Health Survey was used. Those nursing students pursuing a master degree/master of philosophy/doctoral degree were also excluded in this study.

### 2.4. Ethical Considerations

The study was approved by the Human Subjects Ethics Committee and the Institutional Review Board of a local university in Hong Kong (Reference No: HSEARS20160319001). Since some survey questions were sensitive, a letter explaining the purpose, aims and objectives of the study was attached to the front page of the survey. Voluntary participation, anonymity and confidentiality were emphasized. A telephone directory of professional helplines was provided in the survey.

### 2.5. Data Collection Tools and Measurements

Socio-demographic and other lifestyle factors were obtained via a self-reported self-administrative survey. Respondents were asked to assess the truth or otherwise to their own situation over the past week of the following sentences, according to a five-point Likert scale (0: Never; 1: Rarely; 2: Occasionally; 3: Always; 4: All the time). Six questions were asked, as follows:

(1) I ate at least one hot, balanced meal a day
(2) I slept 7–8 h for at least 4 nights
(3) I exercised moderately at least twice
(4) I found time for entertainment at least once
(5) I kept up hobbies (like gardening or playing music)
(6) I had some quiet time to myself every day

#### Depression Anxiety Stress Scale 21 (DASS 21)

We used the validated Chinese version of the Depression Anxiety Stress Scale 21 (DASS 21). This reliable psychological instrument has 21 items in three domains. Each domain comprises seven items assessing three dimensions of mental health symptoms: depression, anxiety and stress. Respondents were required to indicate the presence of these symptom(s) over the past week on a four-point Likert scale scoring from 0 to 3 (0: did not apply at all over the last week, 1: applied to some degree, or some of the time; 2: applied a considerable degree, or a good part of time; 3: applied very much or most of the time). The more severe the symptoms in each dimension, the higher the subscale scores. The instrument is frequently used in clinical and non-clinical samples [6,10,11,12,13] and has well-established psychometric properties in reliably measuring depression, anxiety and stress (at a Cronbach’s alpha of 0.83, 0.80 and 0.82, respectively) in China [14]. The Cronbach’s alphas for each subscale in the Chinese DASS 21 were also comparable to the English version of the DASS 21 [15]. DASS 21 is also taken to yield good estimates of internal consistency for original scale scores (range = 0.82–0.97) [10,16]. The instrument is judged capable of differentiating between depression, anxiety and stress [10,17,18,19,20]. In our study, scores from each dimension were summed up and categorized as “normal”, “mild”, “moderate”, “severe” and “extremely severe” according to the DASS manual [10].

### 2.6. Statistical Analysis

Lifestyle scores were categorized into dichotomous responses (yes/no) before being entered into a logistic regression. Depression, anxiety and stress scores were categorized into a dichotomous response (yes/no) before submitted to univariate analysis. Participants with a cut-off score of ≥10 in depression, ≥8 in anxiety and ≥15 in stress dimension were considered as having these disorders as referenced by the DASS manual [10] (Table 1). Statistical analysis was performed using SPSS Version 23.0 for the Windows platform (SPSS Inc., Chicago, IL, USA). Prevalence estimates (%) were presented at 95% confidence intervals (95% CI) calculated from the Standard Error (SE).

Univariate analysis derived mean values, standard deviations (SD), frequencies (n) and proportion percentages (%) from categorical and continuous variables. Bivariate and multivariate analyses then measured the strength of the associations between variables and sought to identify significant correlates of depression, anxiety and stress. All tests were two-tailed with the level of statistical significance defined as *p* < 0.05. Results were presented as odds ratio (ORs) and as 95% confidence intervals (95% CI).

## 3. Results and Discussion

A total of 1270 nursing students registered in the Baccalaureate Degree in nursing and Baccalaureate Degree in mental health nursing. A total of 661 participants (female = 479) completed the survey, at a 52.6% response rate.

### 3.1. Socio-Demographic, Clinical and Other Characteristics of the Sample Population

The majority of the respondents were female (72.5%, *n* = 479) and were currently in Year 1 to Year 3 (98%, *n* = 647) of their baccalaureate studies. Only a fraction of respondents were in Year 4 (2%, *n* = 14). The mean age was between 18 and 22 years old (SD ± 0.34). All respondents were single. Ninety-seven percent (*n* = 644) lived with family members or others, and 3% (*n* = 17) alone. A total of 68.2 % (*n* = 451) were in general nursing and 31.8% (*n* = 210) in mental health nursing. Less than 30% of participants had some religious faith. Nearly 65% reported financial difficulty (*n* = 429) although only a very small proportion of these were in debt (5%, *n* = 33). Approximately 5%–7% of participants had experienced a past-year relationship crisis with family members, romantic partners or peers. Around 40%–87% were able to maintain a healthy lifestyle, meaning they kept up a balanced diet, exercised, took in some entertainment, kept up hobbies, slept adequately and could have some quiet time by themselves. A relatively low percentage suffered from past-year chronic ill-health (5%, *n* = 33). Fewer than 2% (*n* = 12) self-reported a psychiatric disorder, while 8.5% (*n* = 56) of respondents reported a family history of psychiatric disorder. Only five respondents were current smokers and less than 14% (*n* = 92) were current drinkers. Four respondents used drugs illicitly and approximately 4% gambled. Most respondents perceived their physical and mental health as good (96.2% and 73.7%, respectively) (Table 2).

#### 3.1.1. Depression, Anxiety, Symptoms of Stress and Correlates

Overall, the prevalence of moderately to extremely severe levels of depression, anxiety and symptoms of stress among this cohort came in at 24.3%, 39.9% and 20.0%, respectively. Female nursing students were more likely to report anxiety and stress symptoms, while male students were more likely to report depression than their classmates. Nevertheless, gender was found to be statistically insignificant in predicting depression, anxiety and stress. Age was also not statistically significant in depression and stress, although, interestingly, it did seem significantly correlated with anxiety. The youngest age group (18–22 years) was more likely to report anxiety than the older groups (23–27 years and 28–30 years) (Table 3, Table 4 and Table 5).

On bivariate analysis using binary logistic regression, financial problems; a lack of exercise, entertainment, hobbies, and quiet time; sleep problems; and poor self-perceived physical health were significant correlates of depression, anxiety and symptoms of stress. Clinical specialty and a lack of balanced diet further emerged as significantly correlated with depression and anxiety, while stress was significantly associated with year of study and self-perceived mental health (Table 3, Table 4 and Table 5).

#### 3.1.2. Depression and Correlates

Depression was found to be significantly associated with year of study; clinical specialty; financial difficulties; relationship crises with family and peers; lifestyle factors including a lack of balanced diet, exercise, entertainment, hobbies, and quiet time; sleep problems; and self-perceived physical and mental health. Year 2 students were 4.7 times (crude odds ratio (cOR) 4.69, 95% CI 1.02–21.66) more likely than Year 4 students to report depression, with Year 1 students coming next (cOR 3.04, 95% CI 0.66–13.98) ahead of Year 3 (cOR 2.88, 95% CI 0.63–13.12). General nursing students were 1.7 times more likely to report depression than mental health students (cOR 1.70, 95% CI 1.19–2.44). Students in financial difficulty were 2.3 times (cOR 2.26, 95% CI 1.58–3.24) more likely than those without to experience depressive symptoms. Students who had been through a family crisis were 2–3 times more likely to report depression than those who had not (cOR 3.10, 95% CI 1.48–6.51 and cOR 2.18, 95% CI 1.07–4.46). Poor lifestyle habits including a lack of balanced diet, exercise, entertainment, hobbies, time alone and sleep problems were also significant correlates of depression (all *p*s < 0.05, cOR ranged from 0.4 to 1.6). Students who perceived themselves having poor physical and mental health were 0.4 times and 27 times more likely to report depression than those with good self-perceived physical and mental health (Table 3).

#### 3.1.3. Anxiety and Correlates

Age, lifestyle factors and self-perceived physical health were significantly correlated with anxiety. Nursing students were divided into three age groups (1: 18–22; 2: 23–27; and 3: 28–30). The youngest group was more apt to report anxiety than the other two. Notably, the second group (those aged 23–27) were 60% less likely to experience symptoms of anxiety than the youngest (cOR 0.408, 95% CI 0.233–0.72). General nursing students were 1.8 times more likely to report anxiety than mental health students (cOR 1.840, 95% CI 1.32–2.57). Students in financial difficulty were 2.1 times more likely to experience anxiety symptoms than those without (cOR 2.096, 95% CI 1.51–2.91). Lifestyle factors including poor diet, sleep or exercise as well as a lack of hobbies, leisure activities or quiet time were also significantly associated with anxiety. Students with poor lifestyles were more likely to report anxiety than those with a healthy lifestyle. Students seeing their physical and mental health as poor were, respectively, 3.4 times and 2.9 times more likely to experience anxiety than those with good self-perceived physical and mental health (Table 4).

#### 3.1.4. Stress and Correlates

Stress was significantly associated with year of study, academic failure, financial difficulty, a lack of sleep/exercise/entertainment/hobbies/quiet time. Year 2 students seemed to report more symptoms of stress than Year 1, Year 3 and Year 4 students (cOR 1.368–2.444). Students who had failed in tests/examinations in the past year were 1.7 times (cOR 1.67, 95% CI 1.05–2.66) more likely to experience stress than those who had passed. Students in financial difficulty were 1.8 times (cOR 1.79, 95% CI 1.25–2.56) more likely to report stress than those without money worries. Bad lifestyles, in the sense of a lack of sleep, exercise, entertainment, hobbies or alone time, led to stress among nursing students (by cOR 0.36–1.44, 95% CI 0.24–2.29) compared with healthy-lifestyle students. Students with poor self-perceived physical and mental health were 3.3 times and 8.7 times (cOR 3.27, 95% CI 1.44–7.41 and cOR 8.73, 95% CI 2.44–31.27), respectively, more likely to report symptoms of stress than those students with good self-perceived physical and mental health (Table 5).

All independent variables with a *p* value of <0.25 in the bivariate analysis were taken by the study as important risk factors for depression, anxiety and symptoms of stress and entered into multivariate logistic regression. Our choice of cutoff point (*p* < 0.25) for selecting potentially influential variables was based on an extensive literature review and followed Hosmer and Lemeshow’s recommendation to avoid leaving out potentially important covariates that had failed to be significant in univariate analysis. At the same time, this cutoff was used to screen out those variables of questionable importance [21]. A forward likelihood ratio (LR) was used to identify variables that could be plausibly associated with depression, anxiety and stress in the separate models.

#### 3.1.5. Multivariate Analyses

Multicollinearity (i.e., variance inflation factor (VIF)) in depression, anxiety and stress were examined. The VIF in three dimensions revealed the score of <2, suggesting that all independent variables were not strongly correlated with the dependent factors.

In the final model, eight variables—year of study, clinical specialty, financial difficulty, relationship crisis with family, sleep problems, levels of physical activity, a lack of entertainment and self-perceived mental health—emerged as significant correlates of depression (Table 6). The strongest correlate was self-perceived mental health, which had an adjusted odds ratio (aOR) of 37.46 times, followed by year of study (aOR 3.4) and relationship crisis with family (aOR 3.1). General nursing students with financial difficulty were 2.1 times and 2.7 times, respectively, more likely than those mental health students without financial difficulty to experience depression. Students with sleep problems and no leisure activities like watching TV were twice as vulnerable to depression as those taking time out. Inactive students were 1.6 times more likely to have depressive symptoms than active.

For anxiety, clinical specialty, money and sleep problems, poor diet, a lack of entertainment and self-perceived mental health remained significant predictors in the final model (Table 6). Self-perceived mental health was the strongest correlate (aOR 2.84), followed by financial difficulties (aOR 2.25) and clinical specialty (aOR 2.11). Anxiety was 2.8 times more likely in respondents reporting poor self-perceived mental health, 2.3 times more likely in students with financial difficulty and two times more likely in general nursing students. Students not allowing time for entertainment were twice as likely to report anxiety as those taking time out at least once a week. Individuals eating badly and with sleep problems were 1.8 times and 1.5 times more likely to experience anxiety, respectively.

Poor self-perceived mental health was the strongest predictor of stress (Table 6), with an adjusted OR of 8.29 (95% CI 2.20–31.41), followed by a daily lack of quiet time (aOR 1.97). General nursing students were 1.6 times more likely to experience symptoms of stress than mental health nurses. Respondents with financial difficulties, sleep problems and a schedule meaning no weekly time for entertainment were 1.9 times, 1.7 times and 1.6 times, respectively, more likely to report stress.

There was also a significant correlation between depression, anxiety and symptoms of stress (all *p*s < 0.001, two-tailed; *r* = 0.581 for depression and anxiety, *r* = 0.599 for depression and stress, *r* = 0.581 for anxiety and stress).

### 3.2. Discussion

Our overall estimated prevalence of moderate to extreme severe levels of depression, anxiety and symptoms of stress among baccalaureate nursing students in Hong Kong is of figures of 24.3%, 39.9% and 20.0%, respectively. We found that male nursing students suffered more prevalently from depression and stress than their female classmates. Female nursing students, however, reported greater symptoms of anxiety than male students. Nevertheless, gender was not a statistically significant correlate in these prevalence estimates. Our results were similar to previous studies [12,22,23,24,25,26,27,28]. Nearly a decade ago, Wong et al. [6] conducted a large scale web-based survey of 7915 first-year tertiary education students in Hong Kong using the 42-item Depression Anxiety Stress Scales. Depression, anxiety and stress levels of moderate severity or above were found at incidences of 21%, 41% and 27%, respectively. Our prevalence estimates of depression on Year 1 students were higher (22.5%) than Wong’s while our respondents’ levels of stress were significantly lower (19%); meanwhile, the anxiety levels were comparable (40.1%). Wong et al. also found that female first-year students had significantly higher anxiety and stress scores and male students had significantly higher depression scores than female.

A recent large-scale epidemiological Mental Morbidity Survey in Hong Kong (*n* = 5719, aged between 16 and 75 years) suggests that the weighted prevalence for past-week Common Mixed Mental Disorders (CMD) stands at 13.3% (95% CI 12.40–14.20), with the most frequent reported condition being mixed anxiety and depressive disorder [29]. Our prevalence estimates of depression and anxiety among students comes in at almost two and three times higher than for broader Hong Kong residents.

A cross-sectional study of 506 Malaysian university students aged between 18 and 24 yielded prevalences of moderate to extreme depression, anxiety and stress of 37.2%, 63% and 23.7%, respectively [2]. The authors found no ground for considering gender a correlate of depression or anxiety; female students had significantly higher mean scores of stress than males, however [2]. Shamsuddin also found older students (20–24 years) more likely to be depressed, anxious and stressed than a younger age group (18–19 years). Another cross-sectional study conducted by Bayram and Bilgel [3] on 1617 university students aged between 17 and 26 years in Turkey found depression, anxiety and moderate to severe stress levels of 27.1%, 47.1% and 27%. Anxiety and stress scores were higher among female students. Our prevalence estimates of depression, anxiety and stress symptoms come in lower than Shamsuddin’s and Bayram and Bilgel’s.

Our findings, however, differed more markedly from those of recent prevalence study conducted by Song et al. [3] on 988 Beijing and 802 Hong Kong students. Using the Center for Epidemiologic Studies Depression Scale (CES-D), 36.1% of Hong Kong male students reported a CES-D score of ≥16, 13.4% had scores of ≥25, and 50.7% of Hong Kong female students reported a CES-D score of ≥16, with 21.3% having scores of ≥25. Female students in Hong Kong apparently had significantly higher depression scores than male students (χ^2^ = 15.97, df = 2, *p* < 0.001). There was no statistically significant gender difference in the CES-D scores among the Beijing university freshmen (χ^2^ = 3.101, df = 2, *p* = 0.212). The mixture of Western with Chinese socio-cultural norms and beliefs may contribute to the higher rate of depression among Hong Kong freshmen. Song’s findings importantly suggest an association between psychosocial and environmental factors and depression.

Gender differences as they relate to patterns of psychiatric morbidity may also have an effect on young men and women’s choices of university course [3,30]. Nursing is historically a predominantly female profession. Increasing numbers of men, however, have entered the nursing workforce in recent decades, narrowing the gender gap. Past research has rarely investigated whether gender is a significant correlate in differences in levels of depression, anxiety or stress among nurses. Little is then known on whether male nurses are at higher risk of developing psychiatric morbidity than female. Research consistently reports a higher female prevalence of depression, anxiety and stress symptoms, apparently indicating greater psychological disturbance [31] and distress [32] among women. Male undergraduates, meanwhile, tend to report higher depression rates [33]. This gender differential in morbidity may be attributable to biopsychosocial factors such as gendered social roles [4,34,35]. Researchers seem to have found no consensus on gender as a factor in depression, anxiety and stress, meaning it is difficult to draw conclusion from the apparently gendered distribution of forms of psychiatric morbidity in our study.

#### 3.2.1. Year of Study

In our bivariate analysis, we specifically found that Year 2 students seemed to be more depressed (*p* = 0.05, 95% CI 1.02–21.66) (Table 3) and stressed than Year 4 students, although for stress this was not statistically significant (*p* = 0.18, 95% CI 0.67–9.12) (Table 5). Year 2 students were also more depressed, anxious and stressed than freshmen. We also found an inverse relationship between year of study and depression, anxiety and stress (Table 3, Table 4 and Table 5).

We speculate this may arise as a result of the School of Nursing curriculum design. Freshmen are not required to undertake any clinical practicum. Exemption from the clinical practicum may relieve first years of some depression, anxiety and stress. Students from Year 2 onwards commence their first clinical placement in various hospitals. Placement may be acutely stressful, as can the double workload of book learning and clinical practice [36]. Nonetheless, as students gradually adapt to the clinical environment, their levels of depression, anxiety and stress may fall.

Burnard’s findings and our speculations gain support from recent research by Jimenez et al. [37] who find that 357 nursing students taking diplomas in Spain are more stressed, on average, by clinical than academic or external factors. Psychological symptoms are frequent in these students than physiological. Although students in all years of study reported a moderate level of stress, more experienced nursing students reported more academic stress than novices. Year 2 students were more vulnerable to somatic anxiety symptoms than those in Years 1 and 3. Our findings were further consistent with Bayram and Bilgel [3,38], Tomoda et al. [38] and Dyson and Renk [39] and Jimenez et al [37] in that respondents in Year 1 and 2 students reported depression, anxiety and stress more often Year 3 and 4 students (Table 3, Table 4 and Table 5).

#### 3.2.2. Clinical Specialty

Depression, anxiety and stress were significantly associated with clinical specialty. This study’s nursing students divided into two main streams: (1) general nursing; and (2) mental health nursing. In the multivariate analyses, general nursing students were 2.1 times, 2.1 times and 1.6 times more likely to experience depression, anxiety and symptoms of stress than mental health students (all *p*s < 0.001, aOR 2.13, 95% CI 1.41–3.23; aOR 2.11, 95% CI 1.48–3.01; aOR 1.62, 95% CI 1.11–2.38), respectively. Interestingly, in the authors’ recently published epidemiological data examining the weighted prevalence of depression, anxiety and symptoms of stress among qualified nurses in Hong Kong, general nurses were also found to have a significantly higher level of psychiatric morbidity than mental health nurses [40]. At present, few studies investigate the association between clinical specialty and psychiatric morbidity among nursing professionals. Mental health students are taught about various types of psychiatric disorders, signs and symptoms and treatments throughout their five-year curriculum, as well as receiving wide exposure to practice in different mental health settings. Compared to general nursing students, mental health students might well have greater theoretical and clinical knowledge of mental health. This study’s cross-sectional design means it cannot disentangle causal links between clinical specialty and psychiatric symptoms. Longitudinal or prospective cohort studies measuring levels of depression, anxiety and stress symptoms throughout the transitional period from studying medicine to qualifying could reflect trends in mental health status in nurse professionals.

#### 3.2.3. Relationship Crisis with Family Members

Some research suggests that students experiencing family problems may suffer at school. Family crises may exacerbate students’ risk of depression and affects their physical [41] and mental health [22]. In our sample, a small fraction of students had gone through a relationship crisis with their family in the last 12 months (4.7%, *n* = 31). We found these crises to correlate significantly with depression in bivariate and multivariate analyses. Such students were 3.1 times more likely to report more depression than those without (cOR 3.05, 95% CI 1.35–6.88).

#### 3.2.4. Financial Difficulty

Financial difficulty was another significant correlate of depression, anxiety and stress in the multivariate analyses. Students in financial difficulties were 2.6 times, 2.3 times and 1.9 times more likely to experience depression, anxiety and stress than those without (all *p*s < 0.001, aOR 2.6, 95% CI 1.78–3.93; aOR 2.3, 95% CI 1.60–3.18; aOR 1.88, 95 CI 1.29–2.74, respectively). Yusoff et al. [42] found that the level of stress experienced by students corresponded to family household incomes. Students from lower socio-economic backgrounds faced financial difficulties; students from middle income groups were struggling to fulfill their own and others’ expectations, and students of higher socio-economic status had the money to meet their needs. Other researchers echoed Yusoff’s finding that higher family income was inversely associated with a lower prevalence of depression [7,22,24,25,26,43,44,45,46]. One recent US study found that students characterized by positive signs of anxiety disorder had current financial struggles [24]. Andrews and Wilding [47] concur that financial vulnerability may exacerbate depression, anxiety and stress among university students [47].

It is not uncommon for socially and economically deprived undergraduates in Hong Kong to work part-time according to out-of-class schedules to subsidize their living costs and ease the burden on their families. This will affect students’ studying pattern, making it harder for them to maintain a healthy lifestyle—to exercise, watch entertainment and keep up hobbies. These part-time workers may have serious concerns over their academic performance, disposing them to anxiety, stress and depression [42].

#### 3.2.5. Poor Lifestyle—Imbalanced Diet, a Lack of Exercise/Sleep

Researchers have recently identified three lifestyle factors (diet, exercise, sleep) that play a vital role in the etiology, progression and treatment of depression [48]. For example, the consumption of fish, vegetables, olive oil and cereal correlates negatively with the severity of depressive symptoms in elderly men and women [49]. Research on adolescents [50] and poor older people [51] offers evidence of a link between diet quality and depression. A high intake of fast food (hamburgers, sausages, and pizza) and processed foodstuffs (muffins, doughnuts, and croissants) is associated with an increased risk of depression up to six years later [50].

#### 3.2.6. Lack of a Balanced Diet

Fewer than 15% of our respondents failed to eat one hot, balanced meal a day (13.3%, *n* = 88). Nevertheless, poor diet was a significant correlate of anxiety in bivariate and multivariate analyses. It is believed healthy food consumption largely depends on individuals’ financial circumstances [52,53,54,55]. Few studies look into the link between university students’ financial circumstances and the likelihood of their maintaining a balanced diet. The assumption seems reasonable that poorer students may find it harder to eat well, or may sometimes eat smaller or less nutritious meals on account of lacking funds [56]. Nursing students, though, should know more about others concerning the importance of diet in maintaining good physical health. This knowledge, if students are too poor to buy good food, may itself precipitate anxiety.

#### 3.2.7. Physical Inactivity

In the multivariate analyses, students who did not exercise at least once a week were 1.6 times (cOR 1.63, 95% CI 1.09–2.43) more likely to experience depression than those who did. Our findings were comparable to Feng and coworkers’ [57], whose study investigated the independent and interactive associations of physical activity (PA) and screen time (ST) with depression, anxiety and sleep quality for 1106 Chinese university freshmen. Results showed that high PA and low ST were independently associated with a lower risk of poor sleep (OR 0.48, 95% CI 0.30–0.78) and depression (OR 0.67, 95% CI 0.44–0.89). The American Academy of Pediatrics recommends children and adolescents spend <2 h/day of ST [58]. Excessive ST has been associated with obesity [59], unfavorable blood lipids, backache, headache [60] and poor school performance [61]. Nevertheless, university students may spend long hours looking at computer screens [62], which means they exercise less.

#### 3.2.8. Sleep Problems

Fewer than 30% (*n* = 177) of our respondents had not slept for 7–8 h 3–4 nights a week. Even so, problems sleeping emerged as a significant correlate of depression, anxiety and stress in the multivariate analyses. Results indicate that respondents with sleep problems were 2 times (aOR 2.0, 95% CI 1.36–3.05), 1.5 times (aOR 1.5, 95% CI 1.01–2.19) and 1.7 times (aOR 1.7, 95% CI 1.17–2.53) more likely to experience depression, anxiety and stress than those without. Are these sleep disruptions owing to study-related factors or to factors pertaining to respondents’ personal circumstances?

Some authors [63] suggest an association between poor sleep and depression. Sleep problems precede an episode of depression in 40% of cases. Individuals with persistent sleep problems may be at significantly higher risk of developing depression [64]. It is assumed that depression causes sleep disturbances, but sleep disturbances could be a risk factor for depression [65,66,67,68,69]. That is, upset sleep and depression could be in a mutual cause-and-effect relationship. Insufficient sleep is also associated with poor quality of life, academic performance and mental health [70,71]. Given that the DASS 21 is not a diagnostic instrument in psychiatry and that psychiatric symptoms were only measured for one week and by self-report in this study, it is not possible to examine whether respondents’ poor sleep was the precursor to depression in specific cases without validation by structured clinical interviews.

Nonetheless, in a meta-analysis conducted by Baglioni et al. [72], non-depressive individuals with sleep problems were predicted to be under twice of risk of developing depression than those sleeping satisfactorily. Nevertheless, augmenting antidepressant medication with a symptom-focused cognitive-behavioral therapy for insomnia (CBTI) may enhance treatment outcomes in patients with co-morbid major depression and insomnia [73]. Patients receiving CBTI experienced greater remission rates for both depression (61.5% vs. 33.3%) and insomnia (50.0% vs. 7.7%) compared to a control treatment group. Some authors also suggest the value of mindfulness-based cognitive therapy in treating insomnia symptoms and thereby relieving depression, anxiety and sleep problems in patients with anxiety disorder [74].

#### 3.2.9. Lack of Quiet Time

Only a fraction of students (*n* = 17) in our sample live alone. The vast majority (97.4%, *n* = 644) live with family members or in shared accommodation. Living in a shared housing may offer some social support to students while also diminishing the time students can have by themselves, especially if they are subject to distraction [75]. Dissatisfaction with one’s living environment can induce stress and threaten well-being [76]. Nursing students reporting a lack of quiet time on a daily basis are almost twice as likely to experience symptoms of stress as those finding time for themselves alone (cOR 1.97, 95% CI 1.25–3.11).

#### 3.2.10. Lack of Entertainment

A lack of entertainment (at least once a week) was found to be a significant correlate of depression and anxiety among respondents. Respondents not watching or partaking in entertainment were 2.1 times and two times more likely to experience depression and anxiety than those who did. Recent research has underscored how leisure activities arouse positive emotions, promote self-efficacy, increase competency, and act as buffers for stress [77,78]. Given nursing students’ heavy study burden, they may be especially in need of forms of recreation and relaxation. Through entertainment, nursing students may regain a sense of mastery and self-control, boost their self-esteem, reinforce their relationships and experience periods of happiness before they return to studying [79,80]. Some research suggests some individuals can positively affect their wellbeing through enlightened lifestyle choices [80]. The social and psychological benefits gained from participation in a variety of activities may also reduce social isolation as this is a correlate of depression [77]. These considerations may explain why entertainment stood out as a significant lifestyle factor in the multivariate analyses.

#### 3.2.11. Poor Self-Perceived Mental Health

Poor self-perceived mental health is a significant correlate of depression, anxiety and stress among nursing students in the multivariate analyses (aOR 37.46, 95% CI 4.52–310.30; aOR 2.84, 95% CI 1.06–7.60; aOR 8.29, 95% CI 2.19–31.41, respectively). Thinking oneself ill (for instance, by self-reported somatic complaints) may indicate a subject thinks their quality of life is poor [81]. Psychosomatic complaints and poor perceived quality of life may also be linked with work or study overload and associated stressors. University students have to meet coursework deadlines and try to do well in their studies. Poorer students face a financial as well as an academic burden. Sensitivity to all of these burdens is proven to associate positively with higher depression scores among students [44].

#### 3.2.12. Poor Help-Seeking

In our sample, only a fraction (3.8%, *n* = 25) of respondents sought professional help when depressed, anxious or stressed. They then chose to consult social workers (*n* = 10), general practitioners (*n* = 4), non-government organizations (NGOs) (*n* = 4), telephone helplines (*n* = 1), clinical psychologists/psychiatrists (public) (*n* = 3) and clinical psychologists/psychiatrists (private) (*n* = 3). Apparently, many nursing students with psychiatric symptoms did not perceive a need for professional help, meaning their symptoms went untreated. There are three possible reasons for students not seeking help: (1) they wanted to avoid the stigma associated with psychiatric disorder by dealing with issues themselves or consulting friends; (2) they underestimated the seriousness of their symptoms, possibly thinking stress was part of university life; and/or (3) they lacked the time to go to mental health services. It is thus crucial to identify the barriers for nursing students from seeking help.

## 4. Recommendations

### 4.1. Campus Health

Some behavioral economics research [82] have shown that younger cohorts may respond to subtle interventions that reframe the decision to seek professional help. For example, introducing regular and automatic scheduled health check-ups, mental and physical, as the default for students may lift students’ psychiatric health and destigmatize health issues.

### 4.2. Campus Connectedness

Pidgeon and coworkers’ [83] study of 206 students from the United States, Australia and Hong Kong finds that campus connectedness (CC) moderated the relationship between perceived depression and stress while having no moderating effect on perceived anxiety and stress. Campus connectedness refers to the social connectedness of the university context, designating a student’s sense of psychological belonging to a college environment [84]. Other researchers [84,85,86] also report students undergoing feelings of psychological dislocation in adapting to a new social environment in university.

### 4.3. Mindfulness Meditation

Kang et al. [87] show that mindfulness meditation effectively copes with stress and reduces anxiety among Korean nursing students. Results showed a significant difference in anxiety (F = 6.985, *p* = 0.013) and stress scores (F = 6.145, *p* = 0.020) against a control group (*n* = 20), though not a statistical difference in depression scores. Walach and coworkers’ work [88] on 25 UK college students confirmed a mindfulness meditation-based program on an experiment group (*n* = 14) reduced depression (*z* = 2.097, *p* = 0.04), anxiety (*z* = −2.777, *p* = 0.005) and perceived stress (*z* = 2.356, *p* = 0.02). Gallego et al. [89] endorse Kang’s and Walach’s findings in recommending physical and mindfulness exercises as a means of reducing manifestations of anxiety and stress among junior year students.

### 4.4. Multimedia Interactive Health-Promoting Platform

Interactive multimedia environments may provide a health-promoting platform offering undergraduates opportunities to learn experientially [90]. Jin’s study [91] of 60 American students looked at the effect of incorporating a virtual agent in a computer-aided “entertainment” program, finding that a group taking interactive tests through a virtual agent (the treatment group) enjoyed them more (*t* = 2.25, *p* < 0.05) and found them more educationally valuable than a group taught conventionally (*t* = 2.31, *p* < 0.05). Entertainment-education may also lower stress among students.

## 5. Conclusions

Our study has identified significant predictors of psychosocial disturbance in Hong Kong nursing students. Risk factors include socio-demographic characteristics like age, year of study, the incidence of any family relationship crisis, financial difficulties, and self-perceived mental health; lifestyle factors, such as exercise, lack of time for leisure and quiet time, sleep problems; and work-related factors, including clinical specialty.

Lifestyle factors emerged as significant contributors to poor mental health among nursing students. The implication is that nursing students should make therapeutic lifestyle changes to ensure a good study-life balance and to safeguard their personal well-being. In replications of our study findings, in-depth focus group interviews may be helpful in disentangling the causal relationships we hypothesize between psychiatric symptoms and personal and professional factors. Campus health services can then make a start in formulating effective mental health promoting strategies to maintain the wellbeing of baccalaureate students’ mental health.

## Figures and Tables

**Table 1:** DASS Severity Ratings.

Severity	Depression	Anxiety	Stress
Normal	0–9	0–7	0–14
Mild	10–13	8–9	15–18
Moderate	14–20	10–14	19–25
Severe	21–27	15–19	26–33
Extremely Severe	28+	20+	34+

Source: Lovibond and Lovibond, 1995 [10].

**Table 2:** Frequency distribution of respondents by socio-demographic characteristics and selected variables (*n* = 661).

Demographic Characteristics	Mean	SD	*n*	Percentage (%)
Sex				
Male			182	27.5
Female			479	72.5
Age (years)	18–22	0.34		
18–22			593	89.7
23–27			63	9.5
28–30			5	0.8
Academic years				
Year 1			205	31.0
Year 2			155	23.4
Year 3			287	43.4
Year 4			14	2.1
Specialty				
General nursing			451	68.2
Mental health nursing			210	31.8
Living circumstance				
Living alone			17	21.6
Living with family/others			644	97.4
Religion				
No			479	72.5
Academic failure				
Yes			84	12.7
Financial difficulty				
Yes			429	64.9
Debt (credit card)				
Yes			33	5.0
Relationship crisis with bf/gf				
Yes			47	7.1
Relationship crisis with family				
Yes			31	4.7
Relationship crisis with peers				
Yes			32	4.8
Death of first degree relatives				
Yes			12	1.8
Balanced diet				
No			88	13.3
Sleep Problems				
Yes			484	73.2
Exercise				
No			393	59.5
Entertainment				
No			216	32.7
Hobbies				
No			186	28.1
Quiet time				
No			113	17.1
Chronic illness				
Yes			33	5.0
Psychiatric disorder				
Yes			12	1.8
Family history of psychiatric disorder				
Yes			56	8.5
Gambling				
Yes			28	4.2
Current drinker				
Yes			92	13.9
Smoking status				
Yes			5	0.8
Illicit drug use				
Yes			4	0.6
Self-perceived physical health				
Poor			25	3.8
Self-perceived mental health				
Poor			174	26.3

*n*: Frequency; SD: Standard deviations; bf/gf: boyfriend/girlfriend.

**Table 3:** Frequency distribution of respondents by depression status and socio-demographic characteristics and other selected variables.

Variables	Depression Symptoms		*p*	95% CI	
	Yes (*n*) ^≠^	(%)		Lower Bound	Upper Bound
Sex					
Male **^+^**	70	38.5	-	-	-
Female	162	33.8	0.264	0.57	1.16
Age (years)			0.147		
18–22 **^+^**	215	36.3	-	-	-
23–27	15	23.8	0.052	0.30	1.01
28–30	2	40.0	0.863	0.19	7.07
Academic years *			0.035		
Year 1	69	33.7	0.152	0.66	13.98
Year 2	68	43.9	0.048	1.02	21.66
Year 3	93	32.4	0.172	0.63	13.12
Year 4 **^+^**	2	14.3	-	-	-
Specialty *					
General nursing	175	38.8	0.004	1.19	2.44
Mental health nursing **^+^**	57	27.1	-	-	-
Living circumstance					
Live alone	6	35.3	0.986	0.37	2.76
Living with family/others **^+^**	226	35.1	-	-	-
Religion					
No	170	35.5	0.732	0.74	1.53
Yes **^+^**	62	34.1	-	-	-
Academic failure					
No **^+^**	197	34.1	-	-	-
Yes	35	41.7	0.178	0.86	2.20
Financial difficulty *					
No **^+^**	55	23.7	-	-	-
Yes	177	41.3	0.000	1.58	3.24
Debt (credit card)					
No **^+^**	218	34.7	-	-	-
Yes	14	42.4	0.367	0.68	2.82
Relationship crisis with bf/gf					
No **^+^**	212	34.5	-	-	-
Yes	20	42.6	0.268	0.77	2.56
Relationship crisis with family *					
No **^+^**	213	33.8	-	-	-
Yes	19	61.3	0.003	1.48	6.51
Relationship crisis with peers					
No **^+^**	215	34.2	-	-	-
Yes	17	53.1	0.032	1.07	4.46
Death of first degree relatives					
No **^+^**	228	35.1	-	-	-
Yes	4	33.3	0.897	0.28	3.10
Balanced diet *					
No	40	45.5	0.030	1.05	2.60
Yes **^+^**	192	33.5	-	-	-
Sleep *					
No	90	50.8	0.000	0.28	0.57
Yes **^+^**	142	29.3	-	-	-
Exercise *					
No	153	38.9	0.013	0.47	0.91
Yes **^+^**	79	29.5	-	-	-
Entertainment *					
No	108	50.0	0.000	0.28	0.54
Yes **^+^**	124	27.9	-	-	-
Hobbies *					
No	92	49.5	0.000	0.30	0.61
Yes **^+^**	140	29.5	-	-	-
Quiet time *					
No	56	49.6	0.000	0.32	0.73
Yes **^+^**	176	32.1	-	-	-
Chronic illness					
No **^+^**	222	35.4	-	-	-
Yes	9	27.3	0.342	0.31	1.50
Psychiatric disorder					
No **^+^**	226	34.8	-	-	-
Yes	6	50.0	0.282	0.60	5.87
Family history of psychiatric disorder					
No **^+^**	208	34.4	-	-	-
Yes	24	42.9	0.205	0.82	2.49
Gambling					
No **^+^**	221	34.9	-	-	-
Yes	11	39.3	0.636	0.56	2.62
Current drinker					
No **^+^**	202	35.5	-	-	-
Yes	30	32.6	0.590	0.55	1.40
Smoking status					
No **^+^**	231	35.2	-	-	-
Yes	1	20.0	0.488	0.05	4.14
Illicit drug use					
No **^+^**	231	35.2	-	-	-
Yes	1	25.0	0.674	0.06	5.94
Self-perceived physical health *					
Poor	16	64.0	0.004	0.13	0.67
Good **^+^**	216	34.0	-	-	-
Self-perceived mental health *					
Poor	107	61.5	0.001	3.59	210.40
Good **^+^**	125	25.7	-	-	-

* Variables significant at *p* < 0.05; **^+^** Reference group; **^≠^** DASS Depression Scores of ≥10 (mild, moderate, severe, and extremely severe); bf/gf: boyfriend/girlfriend.

**Table 4:** Frequency distribution of respondents by anxiety status and socio-demographic characteristics and other selected variables.

Variables	Anxiety Symptoms		*p*	95% CI	
	Yes (*n*) ^≠^	(%)		Lower Bound	Upper Bound
Sex					
Male	86	47.3	-	-	-
Female **^+^**	240	50.1	0.513	0.80	1.58
Age (years) *			0.007		
18–22 **^+^**	305	51.4	-	-	-
23–27	19	30.2	0.002	0.23	0.72
28–30	2	40.0	0.614	0.10	3.80
Academic years			0.378		
Year 1	102	49.8	0.315	0.58	5.50
Year 2	84	54.2	0.193	0.68	6.65
Year 3	135	47.0	0.411	0.52	4.89
Year 4 **^+^**	5	35.7	-	-	-
Specialty *					
General nursing	244	54.1	0.000	1.32	2.57
Mental health nursing **^+^**	82	39.0	-	-	-
Living circumstance					
Live alone	8	47.1	0.850	0.35	2.39
Living with family/others **^+^**	318	49.4	-	-	-
Religion					
No	236	49.3	0.967	0.71	1.40
Yes **^+^**	90	49.5	-	-	-
Academic failure					
No **^+^**	281	48.7	-	-	-
Yes	45	53.6	0.405	0.77	1.92
Financial difficulty *					
No **^+^**	87	37.5	-	-	-
Yes	239	55.7	0.000	1.51	2.91
Debt (credit card)					
No **^+^**	308	49.0	-	-	-
Yes	18	54.5	0.539	0.62	2.52
Relationship crisis with bf/gf					
No **^+^**	299	48.7	-	-	-
Yes	27	57.4	0.249	0.78	2.59
Relationship crisis with family					
No **^+^**	306	48.6	-	-	-
Yes	20	64.5	0.088	0.91	4.08
Relationship crisis with peers					
No **^+^**	305	48.5	-	-	-
Yes	21	65.6	0.063	0.96	4.28
Death of first degree relatives					
No **^+^**	332	49.6	-	-	-
Yes	4	33.3	0.272	0.15	1.70
Balanced diet *					
No	58	65.9	0.001	1.38	3.52
Yes **^+^**	268	46.8	-	-	-
Sleep *					
No	107	60.5	0.001	0.38	0.77
Yes **^+^**	219	45.2	-	-	-
Exercise *					
No	209	53.2	0.016	0.50	0.93
Yes **^+^**	117	43.7	-	-	-
Entertainment *					
No	136	63.0	0.000	0.31	0.61
Yes **^+^**	189	42.6	-	-	-
Hobbies *					
No	110	59.1	0.002	0.41	0.81
Yes **^+^**	216	45.5	-	-	-
Quiet time *					
No	69	61.1	0.007	0.37	0.85
Yes **^+^**	257	46.9	-	-	-
Chronic illness					
No **^+^**	309	49.3	-	-	-
Yes	16	48.5	0.929	0.48	1.95
Psychiatric disorder					
No **^+^**	320	49.3	-	-	-
Yes	6	50.0	0.962	0.33	3.22
Family history of psychiatric disorder					
No **^+^**	298	49.3	-	-	-
Yes	28	50.0	0.915	0.60	1.78
Gambling					
No **^+^**	311	49.1	-	-	-
Yes	15	53.6	0.646	0.56	2.55
Current drinker					
No **^+^**	280	49.2	-	-	-
Yes	46	50.0	0.888	0.66	1.60
Smoking status					
No **^+^**	323	49.2	-	-	-
Yes	3	60.0	0.634	0.26	9.32
Illicit drug use					
No **^+^**	324	49.3	-	-	-
Yes	2	50.0	0.978	0.14	7.34
Self-perceived physical health *					
Poor	19	76.0	0.010	1.34	8.61
Good **^+^**	307	48.3	-	-	-
Self-perceived mental health					
Poor	11	73.3	0.072	0.91	9.17
Good **^+^**	315	48.8	-	-	-

* Variables significant at *p* value < 0.05; **^+^** Reference group; **^≠^** DASS Anxiety Scores of ≥8 (mild, moderate, severe, and extremely severe); bf/gf: boyfriend/girlfriend.

**Table 5:** Frequency distribution of respondents by stress status and socio-demographic characteristics and other selected variables.

Variables	Stress Symptoms		*p*	95% CI	
	Yes (*n*) ^≠^	(%)		Lower Bound	Upper Bound
Sex					
Male **^+^**	55	30.2	-	-	-
Female	160	33.4	0.435	0.80	1.68
Age (years)			0.109		
18–22 **^+^**	200	33.7	-	-	-
23–27	13	20.6	0.038	0.27	0.96
28–30	2	40.0	0.768	0.22	7.90
Academic years *			0.029		
Year 1	72	35.1	0.304	0.54	7.35
Year 2	62	40.0	0.183	0.66	9.12
Year 3	78	27.2	0.637	0.37	5.04
Year 4 **^+^**	3	21.4	-	-	-
Specialty					
General nursing	157	34.8	0.067	0.98	2.00
Mental health nursing **^+^**	58	27.6	-	-	-
Living circumstance					
Live alone	5	29.4	0.781	0.30	2.48
Living with family/others **^+^**	210	32.6	-	-	-
Religion					
No	148	30.9	0.148	0.54	1.10
Yes **^+^**	67	36.8	-	-	-
Academic failure *					
No **^+^**	179	31.0	-	-	-
Yes	36	42.9	0.032	1.05	2.66
Financial difficulty *					
No **^+^**	57	24.6	-	-	-
Yes	158	36.8	0.001	1.25	2.56
Debt (credit card)					
No **^+^**	202	32.2	-	-	-
Yes	13	39.4	0.389	0.67	2.81
Relationship crisis with bf/gf					
No **^+^**	194	31.6	-	-	-
Yes	21	44.7	0.068	0.96	3.19
Relationship crisis with family					
No **^+^**	200	31.7	-	-	-
Yes	15	48.4	0.058	0.98	4.16
Relationship crisis with peers					
No **^+^**	200	31.8	-	-	-
Yes	15	46.9	0.080	0.93	3.87
Death of first degree relatives					
No **^+^**	211	32.5	-	-	-
Yes	4	33.3	0.952	0.31	3.49
Balanced diet					
No	35	39.8	0.121	0.91	2.29
Yes **^+^**	180	31.4	-	-	-
Sleep *					
No	80	45.2	0.000	0.33	0.67
Yes **^+^**	135	27.9	-	-	-
Exercise *					
No	144	36.6	0.006	0.44	0.88
Yes **^+^**	71	26.5	-	-	-
Entertainment *					
No	95	44.0	0.000	0.33	0.66
Yes **^+^**	119	26.8	-	-	-
Hobbies *					
No	82	44.1	0.000	0.35	0.70
Yes **^+^**	133	28.0	-	-	-
Quiet time *					
No	59	52.2	0.000	0.24	0.55
Yes **^+^**	156	28.5	-	-	-
Chronic illness					
No *	200	31.9	-	-	-
Yes	14	42.4	0.211	0.77	3.20
Psychiatric disorder					
No **^+^**	208	32.0	-	-	-
Yes	7	58.3	0.066	0.93	9.46
Family history of psychiatric disorder					
No **^+^**	195	32.2	-	-	-
Yes	20	35.7	0.595	0.66	2.07
Gambling					
No **^+^**	206	32.5	-	-	-
Yes	9	32.1	0.965	0.44	2.21
Current drinker					
No **^+^**	184	32.3	-	-	-
Yes	31	33.7	0.796	0.67	1.70
Smoking status					
No **^+^**	215	32.8	-	-	-
Yes	0	0	0.999	-	-
Illicit drug use					
No **^+^**	214	32.6	-	-	-
Yes	1	25.0	0.749	0.07	6.67
Self-perceived physical health *					
Poor	15	60.0	0.005	1.44	7.41
Good **^+^**	200	31.4	-	-	-
Self-perceived mental health *					
Poor	12	80.0	0.001	2.44	31.27
Good **^+^**	203	31.4	-	-	-

* Variables significant at *p* < 0.05; **^+^** Reference category; **^≠^** DASS Stress Scores of ≥15 (mild, moderate, severe, and extremely severe); bf/gf: boyfriend/girlfriend.

**Table 6:** Multiple logistic regression model predicting depression, anxiety and stress symptoms among Hong Kong nurses.

Variable	Categories	B	*p*-Value	aOR	95% CI	
					Lower Bound	Upper Bound
**Depression**						
Constant		−3.363	0.000			
Academic years			0.032			
	Year 1	0.551	0.502	1.735	0.35	8.68
	Year 2	1.222	0.137	3.395	0.68	17.00
	Year 3	0.830	0.306	2.293	0.47	11.22
	Year 4 **^+^**	-	-	-	-	-
Specialty	General nursing	0.756	0.000	2.130	1.41	3.23
	Mental health nursing **^+^**	-	-	-	-	-
Financial difficulty	No **^+^**	-	-	-	-	-
	Yes	0.973	0.000	2.646	1.78	3.93
Relationship crisis with family	No **^+^**	-	-	-	-	-
	Yes	1.116	0.007	3.051	1.35	6.88
Maintain 7–8 h sleep 3–4 times per week	No	0.711	0.001	2.035	1.36	3.05
	Yes **^+^**	-	-	-	-	-
Physical activity level	Inactive	0.486	0.017	1.626	1.09	2.43
	Active **^+^**	-	-	-	-	-
Entertainment	No	0.731	0.000	2.077	1.41	3.06
	Yes **^+^**	-	-	-	-	-
Self-perceived mental health	Poor	3.623	0.001	37.455	4.52	310.30
	Good **^+^**	-	-	-	-	-
**Anxiety**						
Constant		−1.508	0.000	0.221		
Specialty	General nursing	0.748	0.000	2.112	1.48	3.01
	Mental health nursing **^+^**	-	-	-	-	-
Financial difficulty	No **^+^**	-	-	-	-	-
	Yes	0.812	0.000	2.252	1.60	3.18
Maintain 7–8 h sleep 3–4 times per week	No	0.397	0.044	1.487	1.01	2.19
	Yes **^+^**	-	-	-	-	-
Balanced diet daily	No	0.578	0.026	1.782	1.07	2.96
	Yes **^+^**	-	-	-	-	-
Entertainment	No	0.689	0.000	1.993	1.39	2.85
	Yes **^+^**	-	-	-	-	-
Self-perceived mental health	Poor	1.043	0.038	2.838	1.06	7.60
	Good **^+^**	-	-	-	-	-
**Stress**						
Constant		−2.001	0.000	0.135		
Specialty	General nursing	0.484	0.013	1.623	1.11	2.38
	Mental health nursing ****^+^****	-	-	-	-	-
Financial difficulty	No **^+^**	-	-	-	-	-
	Yes	0.630	0.001	1.877	1.29	2.74
Maintain 7–8 h sleep 3–4 times per week	No	0.540	0.006	1.717	1.17	2.53
	Yes **^+^**	-	-	-	-	-
Entertainment	No	0.467	0.016	1.596	1.09	2.33
	Yes **^+^**	-	-	-	-	-
Quiet time by self daily	No	0.680	0.003	1.973	1.25	3.11
	Yes **^+^**	-	-	-	-	-
Self-perceived mental health	Poor	2.116	0.002	8.294	2.19	31.41
	Good **^+^**	-	-	-	-	-

aOR: adjusted odds ratio; **^+^** Reference group.
